# TRIM29 promotes bladder cancer invasion by regulating the intermediate filament network and focal adhesion

**DOI:** 10.1038/s41388-025-03557-z

**Published:** 2025-09-04

**Authors:** Yin Wang, Nicole A. Jerome, Alan J. Kelleher, Marian L. Henderson, Mark L. Day, Pierre A. Coulombe, Phillip L. Palmbos

**Affiliations:** 1 Department of Internal Medicine, Hematology/Oncology Division, University of Michigan Medical School, Ann Arbor, MI USA; 2 Urology, University of Michigan Medical School, Ann Arbor, MI USA; 3 Cell & Developmental Biology, University of Michigan Medical School, Ann Arbor, MI USA; 4 Dermatology, University of Michigan Medical School, Ann Arbor, MI USA

**Keywords:** Bladder cancer, Focal adhesion, Intermediate filaments

## Abstract

Bladder cancer is a common malignancy whose lethality is determined by invasive potential. We have previously shown that *TRIM29*, also known as *ATDC*, is transcriptionally regulated by TP63 in basal bladder cancers where it promotes invasive progression and metastasis, but the molecular events which promote invasion and metastasis downstream of *TRIM29* remained poorly understood. Here we identify stimulation of bladder cancer migration as the specific role of TRIM29 during invasion. We show that TRIM29 physically interacts with K14+ intermediate filaments which, in turn, regulates focal adhesion stability. Further, we find that both K14 and the focal adhesion protein, ZYX are required for bladder cancer migration and invasion. Taken together, these results establish a role for TRIM29 in the regulation of cytoskeleton and focal adhesions during invasion and identify a pathway with therapeutic potential.

## Introduction

Bladder cancer is the sixth most common cancer in the United States and will cause >17,000 deaths in 2023 [1]. Clinically, bladder cancers are categorized as either non-muscle-invasive (NMI) or muscle-invasive (MI) based on their ability to invade stroma and muscular barriers. NMI bladder cancers are mostly low-grade, multifocal, and frequently recur after surgical removal. Approximately 15% of these NMI bladder tumors will progress to MI bladder cancers. MI bladder cancers in which tumors invade through the basement membrane and migrate into the muscularis propria require more aggressive multimodality therapy with chemotherapy, radiation, and surgery. Despite this aggressive and toxic therapy, up to 50% of patients will have lethal metastatic relapses. Thus, invasive progression determines patient outcomes in bladder cancer.

Despite the clinical importance, the molecular drivers of invasive progression in bladder cancer are poorly understood. We have previously identified Tripartite Motif-containing protein 29 (TRIM29), also known as Ataxia Telangiectasia Group D Complementing (ATDC), as an important driver of bladder cancer initiation and invasive progression [2, 3]. *TRIM29*, along with *KRT14*, is part of the basal gene program which drives tumor progression and metastasis [3]. However, the molecular mechanism by which *TRIM29* impacts bladder cancer invasion was not previously understood.

Cell motility plays a key role in cancer invasion and is regulated by a network consisting of highly coordinated cytoskeleton proteins including actin microfilaments and intermediate filament (IF) proteins such as vimentin and keratins. Keratin 14 (K14), one of the components of IF proteins and a marker of basal epithelium, is upregulated in the leading cells of invading breast tumor and plays an important role in regulating invasion [4]. We previously reported that *TRIM29* and *KRT14* are both upregulated by TP63 in invasive basal bladder cancers [3], but how TRIM29 and K14 interact with the cytoskeleton to coordinate invasion remained unclear.

The focal adhesion complex (FA), which serves as an anchor point for cells to attach to the extracellular matrix (ECM), is one of the main structures connecting the ECM to intracellular cytoskeleton networks including actin microfilaments, microtubules, and intermediate filaments [5, 6]. The FA complex is a dynamic protein complex required for proliferation, migration, and invasion of cancer cells [7, 8]. The FA complex develops on the cytoplasmic side of the cell membrane, where integrin receptors cluster. FA complexes are comprised of scaffold or adapter proteins like paxillin (PXN), which recruit downstream signaling factors, including focal adhesion kinase (FAK) [9]. Phosphorylation/activation of FAK promotes binding of regulatory proteins such as c-Src, vinculin, and talin, leading to polymerized actin assembly and a physical connection between focal adhesion sites and cytoskeleton network. These events eventually enhance cell motility and invasion by regulating dynamic rearrangement of the actin cytoskeleton [10, 11].

In this study, we use 2D and 3D models of migration and invasion to identify the role of TRIM29 in bladder cancer invasion. We find that TRIM29 specifically regulates cell migration by binding to K14+ IF and regulates formation of K14+ IFs in the invadopodia of bladder cancer cells. Further, this function regulates the stability of the FA complex during cancer cell migration and invasion. These results provide a better understanding of the regulation of invasive progression in bladder cancer and identify new potential therapeutic targets to prevent progression to the lethal invasive form of bladder cancer.

## Materials and methods

### Cell lines and culture media

UM-UC5, UM-UC9, UM-UC10, UM-UC13, UM-UC14 and UM-UC15 invasive bladder cancer cells were cultured in DMEM culture media supplied with 4.5 g/L D-Glucose, L-Glutamine (2.5 mM), 110 mg/L Sodium Pyruvate, and 10% FBS (Thermo Fisher Scientific, Waltham, MA). Each cell line is from an invasive tumor and expresses medium to high levels of TRIM29 and K14. All cell lines were fingerprinted and mycoplasma-tested negative. Generation of the isogenic UM-UC5, 9 and 14 *TRIM29*-KO cell lines using CRISPR-Cas9 has previously been published [3].

### Immunoblot

Immunoblot technique was described previously [3]. In short, 25 µg of cell lysates were loaded into a protein gel (Bio-Rad, #4561094) along with protein ladders (Bio-Rad, #1610374) as molecular weight indicators to locate the protein of interest. The samples were separated by electrophoresis at 200 V for about 40 minutes in running buffer (Bio-Rad, #1610772). Proteins were then transferred onto a PVDF membrane (Millipore, #IPVH00010) using a wet transfer system at 85 V for 90 min at 4 °C. The membrane was blocked with 5% non-fat milk in TBST (Tris-buffered saline with 0.1% Tween-20) for 1 h at room temperature and incubated with primary antibody diluted in TBS overnight at 4°C. After washing with TBST, the membrane was incubated with fluorescent secondary antibody for 1 h at room temperature, and protein bands were visualized using a fluorescence imager (Odyssey, LI-COR, Lincoln, NE).

### Antibodies

TRIM29 (sc-166707, Santa Cruz Biotechnology, Dallas, TX), TRIM29 (HPA020053, Sigma-Aldrich, St. Louis, MO), K14 (ab7800, Abcam, Cambridge, UK), K14 (HPA023040, Sigma-Aldrich), Paxillin (#2542, Cell Signaling Technology, Danvers, MA), Zyxin (HPA004835, Sigma-Aldrich), FAK (#610087, BD Transduction Laboratories, Franklin Lakes, NJ), β-Actin (A1978, Sigma-Aldrich), FAM83H (HPA024604, Sigma-Aldrich), mCherry (PA5-34974, Thermo Fisher Scientific), mCherry (600-401-P16, Rockland Immunochemicals, Limerick, PA), HA (#600-401-384, Rockland Immunochemicals), HA (08L100032, MP Biomedicals, Irvine, CA), secondary antibodies for immunofluorescence staining (A11001, A21244, Thermo Fisher Scientific), secondary antibodies for western blotting (#926-68071 and #926-32210, LI-COR).

### Expression vectors and siRNA knockdowns

Plasmids for mCherry-K14 (#55066), HA-ubiquitin (#74218), and GFP (#45561, as vector control) were purchased from Addgene, Watertown, MA. Plasmid for overexpressing FLAG-tagged TRIM29 was designed by our lab and made by VectorBuilder, Chicago, IL. The transfection and siRNA protocols were described previously [3]. Gene knockdown of K14 or TRIM29 was performed by using siRNA against *KRT14* (E-010602-00-0005, Horizon Discovery, Waterbeach, United Kingdom) or *TRIM29* (E-012409-00-0005, Horizon Discovery), respectively. Overexpression of HA-tagged ubiquitin or FLAG-tagged TRIM29 was carried out by using the lentiviral transduction system described previously [3].

### Modified 2D cell migration (Scratch) assay and phase-contrast microscopy

A cover glass-bottomed 4-well chamber (#155382 Nunc Lab-Tek II, Thermo Fisher Scientific) was coated with poly-L-lysine (#4832, Sigma-Aldrich) and type I collagen solution (3 mg/ml, #04902, STEMCELL Technologies, Vancouver, Canada); a cell culture insert (#80209, Ibidi, Gräfelfing, Germany) was placed securely on the coated cover glass. Cells reaching 80% confluence were trypsinized and transferred into the cell culture insert (3.5 × 10^4^ cells/insert chamber). Cells were cultured inside the insert overnight (or until the cells reach 80% or 90% confluence). The insert was removed to start the migration assay. 2D migration was observed and recorded by a LSM800 microscope (Zeiss, Oberkochen, Germany) equipped with time-lapse imaging ability and a climate control chamber. Images were taken at 10-min intervals and then analyzed by ZEN2 software (Zeiss). Cells on the leading edge were selected and tracked manually for calculating the migration velocity.

### Transwell invasion assay

Transwell invasion assays were conducted using 24-well plates with transwell inserts (8-µm pore size; #354480, Corning, Corning, NY). A total of 2.5 × 10⁴ cells suspended in serum-free medium were seeded into the upper chamber, while the lower chamber was filled with medium supplemented with 10% fetal bovine serum as a chemoattractant. After 24 h of incubation at 37 °C, non-invading cells were carefully removed from the upper surface of the membrane using a cotton swab. Invaded cells on the lower membrane surface were fixed with 4% paraformaldehyde (157-4-1L, Electron Microscopy Sciences, Hatfield, PA), stained with 0.1% crystal violet, and quantified under a light microscope.

### 3D tumor spheroid invasion assay

3D tumor spheroid invasion assays were conducted as previously described [12]. In short, bladder cancer cells were grown into spheroids in 6-well ultra-low attachment plates for 24 hours, then embedded into collagen type I matrix (#354236, Corning) for time-lapse live-cell imaging by confocal microscopy.

### Immunoprecipitation

Cells cultured on plates reaching 90% confluence were lysed in buffer (25 mM Tris-HCl pH 7.4, 150 mM NaCl, 1 mM EDTA, 5% glycerol) for immunoprecipitation (IP). For conventional IP, the lysis buffer contained 0.1% NP-40. Cell lysates were centrifuged at 3000 × *g* for 10 min, and then the supernatants were collected. Magnetic beads (10015D, Thermo Fisher Scientific) were coated with TRIM29 antibody (B-2, Santa Cruz Biotechnology, Dallas, TX) for 6 h at 4 °C before adding to cell lysates by following manufacturer’s instructions. Antibody-coated beads and cell lysates were mixed in 4 °C for overnight. Beads were washed with cold PBS twice before adding protein sample loading buffer (#1610747, Bio-Rad, Hercules, CA) and heated at 95 °C for 5 min. Immunoprecipitated samples were used to perform Western blot or mass spectrometry.

### Liquid chromatography-mass spectrometry (LC-MS)

The IP beads were resuspended in 50 µl of 0.1 M ammonium bicarbonate buffer (pH~8). Cysteines were reduced by adding 50 µl of 10 mM DTT and incubating at 45 °C for 30 min Samples were cooled to room temperature and alkylation of cysteines was achieved by incubating with 65 mM 2-chloroacetamide under darkness for 30 min at room temperature. An overnight digestion with 1 µg sequencing grade, modified trypsin was carried out at 37 °C with constant shaking in a thermomixer. Digestion was stopped by acidification and peptides were desalted using SepPak C18 cartridges following manufacturer’s protocol (Waters, Milford, MA). Samples were completely dried by using vacufuge. Resulting peptides were dissolved in 9 µl of 0.1% formic acid/2% acetonitrile solution and 2 µl of the peptide solution were resolved on a nano-capillary reverse phase column (Acclaim PepMap C18, 2 micron, 50 cm, Thermo Fisher Scientific) using a 0.1% formic acid/2% acetonitrile (Buffer A) and 0.1% formic acid/95% acetonitrile (Buffer B) gradient at 300 nl/min over a period of 180 min (2–25% Buffer B in 110 min, 25-40% in 20 min, 40–90% in 5 min followed by holding at 90% Buffer B for 10 min and equilibration with Buffer A for 30 min). Eluent was directly introduced into Q exactive HF mass spectrometer (Thermo Fisher Scientific) using an EasySpray source. MS1 scans were acquired at 60 K resolution (AGC target = 3 × 106; max IT = 50 ms). Data dependent collision induced dissociation MS/MS spectra were acquired using Top speed method (3 seconds) following each MS1 scan (NCE~28%; 15 K resolution; AGC target 1 × 105; max IT 45 ms). Proteins were identified by searching the MS/MS Human Protein Database (20286 entries; reviewed; downloaded on 06/17/2020) and filtered for high confidence proteins using Proteome Discoverer (v2.4, Thermo Fisher Scientific). Search parameters included MS1 mass tolerance of 10 ppm and fragment tolerance of 0.2 Da; two missed cleavages were allowed; carbamidimethylation of cysteine was considered fixed modification and oxidation of methionine, deamidation of asparagine and glutamine were considered as potential modifications. False discovery rate (FDR) was determined using Percolator and proteins/peptides with an FDR of =1% were retained for further analysis.

### Immunofluorescence staining and imaging

Cells were seeded on poly-L-lysine- (#4832, Sigma-Aldrich) and collagen- (3 mg/ml, #04902, STEMCELL Technologies, Vancouver, Canada) coated cover glass. After designated treatment and time of incubation, cell samples were fixed by 4% paraformaldehyde in PBS for 10 min, then permeabilized with 0.5% Triton X-100 in PBS for 5 min. After thoroughly washing with PBS, samples were submerged in blocking solution (PBS containing 5% BSA) for 1 h at room temperature. After blocking, the samples were covered with primary antibody solution (designated primary antibody in PBS, 1:50 dilution) at room temperature for 16 h, followed by washing thoroughly with PBS. The samples were then incubated in secondary antibody solution (1:300 dilution) for 1 h at room temperature and washed thoroughly with PBS before staining for nuclei with Hoechst 33342 (Thermo Fisher Scientific) solution for 5 min at room temperature. Samples were washed thoroughly again with PBS before mounting on glass slides with 30 µl mounting media. Images were obtained with the LSM800 confocal microscope (Zeiss). For co-localization analysis of TRIM29 and K14, pre-formed bladder cancer spheroids were plated onto collagen-coated glass coverslips and allowed to adhere and expand for 48 hours. Immunofluorescence images were captured specifically at the leading edge of migrating cells spreading out from the spheroid to ensure cells were in an active migratory state.

### Realtime imaging of K14± intermediate filaments during cell migration

UM-UC5 or UM-UC14 cells were transfected with mCherry-labeled K14 (#55066, Addgene). Cells were cultured on collagen- (3 mg/ml, #04902, STEMCELL Technologies) coated cover glass chamber slides within a culture insert. After removal of the insert, LSM800 confocal microscope (Zeiss) was used to visualize the area covered by the migrating cells. Time-lapse imaging was conducted at 3-minute intervals using a 63X oil objective.

### Quantification of the number and size of focal adhesion sites

Confluent bladder cancer cells were trypsinized and cultured in ultra-low attachment 6-well culture plate (0.5 × 10^6^ cells/well) for 24 h to form cancer spheroids. Spheroids were transferred to a 12-well plate with one poly-L-lysine- (#4832, Sigma-Aldrich) and collagen- (#04902, 3 mg/ml, STEMCELL Technologies) coated coverslip placed in each well. Spheroids were allowed to attach and expand on coverslip for 48 h at 37 °C with 5% CO_2_ supply_._ Samples were fixed and stained with anti-ZYX or anti-PXN antibodies according to immunofluorescence staining procedures described above. Images were taken by a LSM800 confocal microscope (Zeiss). The cells located on the leading edge of the expanded spheroids were selected manually by using ZEN2 software. The size and number of the ZYX or PXN+ focal adhesion sites, were quantitated using ZEN2. Only focal adhesion sites ≥0.02 µm^2^ were examined. Site number/cell was recorded.

### Realtime analysis of focal adhesion dynamics

*TRIM29* wildtype or KO bladder cancer cells (UM-UC5 and UM-UC14) were transfected with a plasmid expressing mCherry-tagged PXN (#50526, Addgene) before culturing on poly-L-lysine- (#4832, Sigma-Aldrich) and collagen- (3 mg/ml, #04902, STEMCELL Technologies) coated cover glass chamber with culture inserts. The insert was removed to allow cells to migrate for 72 h, and the area of the migration was imaged with a LSM800 confocal microscope (Zeiss). Time-lapse imaging was carried out with 3-min intervals and seven slices of z stack (spanning height of 70 nm) by using 63× oil objective. The quantification of focal adhesion dynamics, including the rate of FA assembly (Ka) and disassembly (Kd), was performed according to previously described methods [13].

### HA-ubiquitination assay

Ubiquitin-HA (#74218, Addgene). was overexpressed in UM-UC14 wildtype (WT) and *TRIM29*-KO cells via lentiviral infection, followed by selection with puromycin for two weeks. Subsequently, cells were transfected with K14-mCherry plasmid (#55066, Addgene), and 48 hours post-transfection, mCherry-positive cells were enriched through fluorescence-activated cell sorting (FACS) and cultured for further experiments. Prior to cell lysis, MG132 (10 µM) was added to the cultures for 6 h to inhibit proteasomal degradation. Cell lysates were prepared, and 1 mg of total protein was subjected to immunoprecipitation (IP).

### Statistical analyses

The statistical tests and number of replicates used are detailed in the text and figure legends. Basic statistical analyses were conducted using Student’s t-test.

## Results

### Loss of TRIM29 blocks bladder cancer migration

**Fig. 1:**
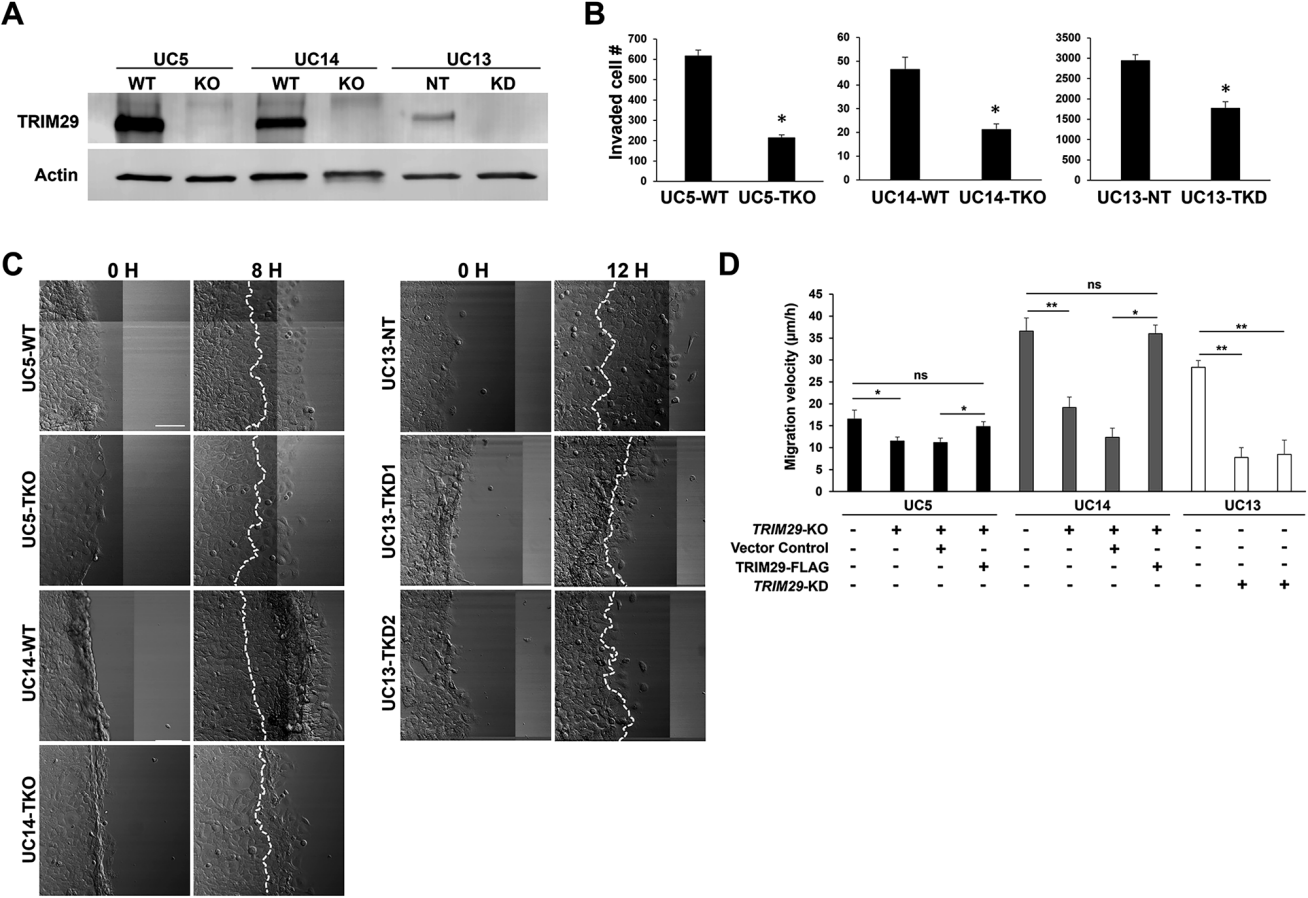
TRIM29 regulates invasion and migration of bladder cancer cells. **A** Knockout (KO) or knockdown (KD) of TRIM29 expression assessed by immunoblot. NT nontargeting siRNA control. **B** KO or KD of TRIM29 decreases invasion in a transwell invasion assay. WT wildtype, TKO *TRIM29*-KO, TKD *TRIM29*-KD. *n* = 6. *p < 0.001 by t-test. **C** Timelapse images of 2D migration assay demonstrate that *TRIM29* knockout (TKO) or knockdown (TKD) decreases migration of human bladder cancer cells. White dashed lines indicate the leading edges at start timepoint. Scale bar = 100 µm. **D** Quantitative analysis of 2D migration assay. Data represent the mean ± STD. n = 50. *p < 0.01, **p < 0.0001, ns = not significant by *t*-test.

The tripartite motif gene, *TRIM29*, is required for invasion in bladder cancer [2, 3] and many other malignancies [14, 15]. Invasion is a complex process that involves degradation of extracellular matrix barriers, cellular detachment and reattachment, and increased motility [16, 17]. To determine which of these aspects of invasion were regulated by TRIM29 in bladder cancer, we examined invasion and migration using transwell, 3D collagen tumor spheroid invasion and modified 2D scratch assays. *TRIM29* was knocked down (*TRIM29*-KD) with shRNA in UM-UC13 or knocked out (KO) with CRISPR-Cas9 in UM-UC14 and UM-UC5 human invasive bladder cancer cell lines (Fig. 1A) [3]. As expected, *TRIM29* knockdown (TKD) in UM-UC13 and *TRIM29* knockout (TKO) in UM-UC5 and UM-UC14 significantly reduced transwell invasion (Fig. 1B) and 3D tumor spheroid invasion (Supplementary Fig. 1). To determine whether TRIM29 was specifically required for cancer cell migration, we performed a modified scratch assay in which cells were cultured on collagen type I-coated cover glass, a scratch was created by insert removal, and individual cell migration velocity was quantified by time-lapse microscopy. *TRIM29*-KO (UM-UC5, UM-UC14) or *TRIM29*-KD (UM-UC13) significantly reduced migration of individual cancer cells (Figs. 1C, D). Re-expression of TRIM29-FLAG in the *TRIM29*-KO cells (UM-UC5 and UM-UC14) rescued the cell migration ability disrupted by knockout of *TRIM29* (Fig. 1D, Supplemental Fig. 2). These results establish that *TRIM29* is required for bladder cancer cell migration during invasion.

### TRIM29 is part of a protein complex including the cytoskeleton and focal adhesion proteins

TRIM29 has previously been demonstrated to exert cellular effects by binding and sequestering proteins and regulating ubiquitination and protein stability [2, 18–21]. We have previously shown that the expression level of TRIM29 is coregulated with the intermediate filament protein, K14, during invasion [3]. To identify the interactome of TRIM29 in bladder cancer and identify the potential mechanism by which TRIM29 promotes bladder cancer migration and invasion, we performed immunoprecipitation (IP) of TRIM29 and liquid chromatography-mass spectrometry (LC-MS) in UM-UC5 and UM-UC9 bladder cancer cell lines to identify proteins present in the immunocomplex with TRIM29. UM-UC5 and UM-UC9 *TRIM29*-KO cells which lack TRIM29 expression were subjected to TRIM29 IP/LC-MS to control for nonspecific pulldown and act as a negative control. We identified 1125 proteins in UM-UC5 and 270 proteins in UM-UC9 which were selectively immunoprecipitated in *TRIM29* WT but not KO samples (Supplementary Table 1). 144 of these proteins were detected in both UM-UC5 and UM-UC9. As expected, TRIM29 was one of the most enriched proteins in both cell lines. To identify the functional classes of the 1125 and 270 proteins selectively co-IP’d with TRIM29 in UM-UC5 and UM-UC9, we subjected each gene list and the 144 gene overlap list to KEGG pathway enrichment analysis. For both UM-UC5 and UM-UC9, there was striking enrichment in genes related to Regulation of Actin Cytoskeleton and Focal Adhesion in our TRIM29 co-IP protein complexes (Supplementary Table 2). Within these pathways, the proteins involved in regulation of focal adhesion and regulation of the actin cytoskeleton have previously been shown to regulate cell adhesion, migration and invasion [22]. These results suggest that TRIM29 forms protein complexes with focal adhesion and cytoskeleton, suggesting a potential means whereby TRIM29 regulates cancer migration and invasion.

### TRIM29 regulates K14 ± IF in invasive and migratory cells

**Fig. 2:**
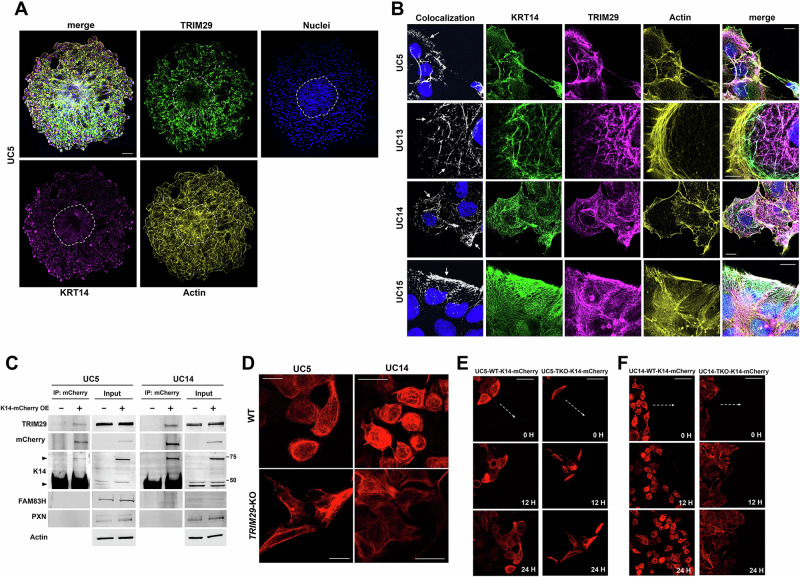
TRIM29 and K14 are upregulated in migratory cells during bladder cancer spheroid invasion. **A** Immunofluorescence images of UM-UC5 spheroids show enriched expression of TRIM29 and K14 as compared to noninvasive cells (center dashed circle, original outline of the spheroid). Scale bar = 100 µm. **B** Colocalization (white arrows) of filamentous TRIM29 and K14 in multiple human bladder cancer cell lines. Scale bar = 10 µm. **C** Co-immunoprecipitation of mCherry-tagged K14 and TRIM29 demonstrate a physical association between K14 and TRIM29. Protein loading for input blots: 20 µg/lane. Arrow heads: endogenous K14 (bottom) and mCherry-tagged K14 (top). **D** *TRIM29-*KO alters distribution of K14-containing IF and cell morphology in migrating cells. Scale bar = 25 µm. **E**, **F** *TRIM29*-KO in UM-UC5 and UM-UC14 results in altered cell morphology and disordered migration in a modified scratch assay. Dashed white arrow = direction of migration. Scale bar = 50 µm. WT wildtype, TKO *TRIM29*-KO.

*KRT14* is transcriptionally regulated by TP63 [23] and is upregulated in invasive leader cells in breast cancer by TP63 [4]. We have previously shown that TP63 regulates transcription of *TRIM29* and *KRT14* in basal bladder cancer, that TRIM29 and K14 are both required for TP63-induced bladder cancer invasion [3]. Since TRIM29 forms a protein complex with IF proteins in bladder cancer cells in our LC-MS screen and regulates keratin distribution in squamous cell carcinoma [24], we hypothesized that TRIM29 might specifically localize to K14-containing IF proteins to regulate migration and invasion. To examine this, we generated bladder cancer spheroids in suspension conditions as previously described and embedded them in collagen on coverslip [3]. Spheroids were cultured for 48 h, fixed and subjected to immunofluorescent staining for TRIM29, actin and K14. Both TRIM29 and K14 were selectively upregulated in the cells in the invasive component of the bladder cancer spheroids (Fig. 2A, region outside of dotted line). These results suggest that TRIM29 is selectively upregulated with K14 in the invading tumor cells.

TRIM29 localizes to filamentous structures and has previously been reported to interact with the IF protein, Vimentin [25]. Because TRIM29 is coregulated with K14, an intermediate filament protein, we hypothesized that TRIM29 might localize to IF proteins during invasion. To determine this, we performed immunofluorescent staining for TRIM29, K14, and actin in multiple bladder cancer cell lines. We chose K14 and not other keratins identified in the LC-MS screen because of our prior data linking TRIM29 function to K14 [3]. TRIM29 and K14 strongly co-localized to the IF structures observed in the invasive cells of multiple bladder cancer cell lines (colocalization between K14 and TRIM29 shown in white, Fig. 2B). Interestingly, although TRIM29 and K14 were always present in contiguous IF structures throughout the cytoplasm, the peripheral, membrane proximal regions in lamellipodia showed the highest overlap (arrows, Fig. 2B). Given that TRIM29 and K14 were selectively upregulated in migratory/invasive cells (Fig. 2A), these results suggest that TRIM29 and K14 might both be present in the same protein complex during migration.

To determine the role of TRIM29 in K14 filament dynamics in migratory bladder cancer cells, we transduced UM-UC5 and UM-UC14 cells with a K14-mCherry fusion protein (Fig. 2C). To determine if K14 and TRIM29 were part of the same protein complex in these cells, we performed co-immunoprecipitation (IP) using an anti-mCherry antibody. We found that IP of mCherry-K14 resulted in co-IP of TRIM29 in UM-UC5 and UM-UC14 cell lines, but not in the control cells lacking K14-mCherry (Fig. 2C). Likewise, IP of TRIM29 also resulted in co-IP of K14-mCherry (Supplementary Fig. 3) establishing that TRIM29 and K14 are part of the same protein complex.

Next, to assess the role of TRIM29 in regulation of K14 dynamics during migration, we observed migrating WT and *TRIM29*-KO UM-UC5 and UM-UC14 cells expressing K14-mCherry (Fig. 2D–F, Supplementary Video 1-4). In *TRIM29* WT cells, the migratory cells formed a rigid, symmetrical, and compact cell structure with filamentous K14 distributed evenly through the cytoplasm (Fig. 2D, Supplementary Video 1 and 3). In contrast, *TRIM29*-KO cells displayed irregular elongated morphologies and K14 did not form regular filamentous structures (Fig. 2D, Supplementary Video 2 and 4). Further, examination of migration in *TRIM29* WT and KO cells using time-lapse imaging demonstrated that while *TRIM29* WT cells showed well organized K14+ filaments and efficient cell migration, *TRIM29*-KO cells lacked organized K14 filaments and demonstrated disordered migration (Fig. 2E, F, Supplementary Video 1–4). Similar results were observed by fixing TRIM29 WT and KO cells and staining for K14 (Supplementary Fig. 4). These results suggest that loss of TRIM29 destabilizes K14+ intermediate filaments, changes the morphology of invasive cells and impairs migratory ability.

### K14 is not a significant target of ubiquitination by TRIM29 in bladder cancer cells

We next sought to determine the mechanism by which TRIM29 regulates K14+ IF during invasion. FAM83H has been proposed to bind to TRIM29 and K14 and to regulate K14 distribution in other types of cancer [24]. We hypothesized that TRIM29 regulation of K14 might involve interaction with FAM83H. Interestingly, while FAM83H was expressed in UM-UC5 and UM-UC14 cancer cells, it did not co-IP with K14 or TRIM29 under all of the tested conditions, suggesting that it may not strongly associate with the TRIM29-K14 complex in these bladder cancer models (Fig. 2C, data not shown).

**Fig. 3:**
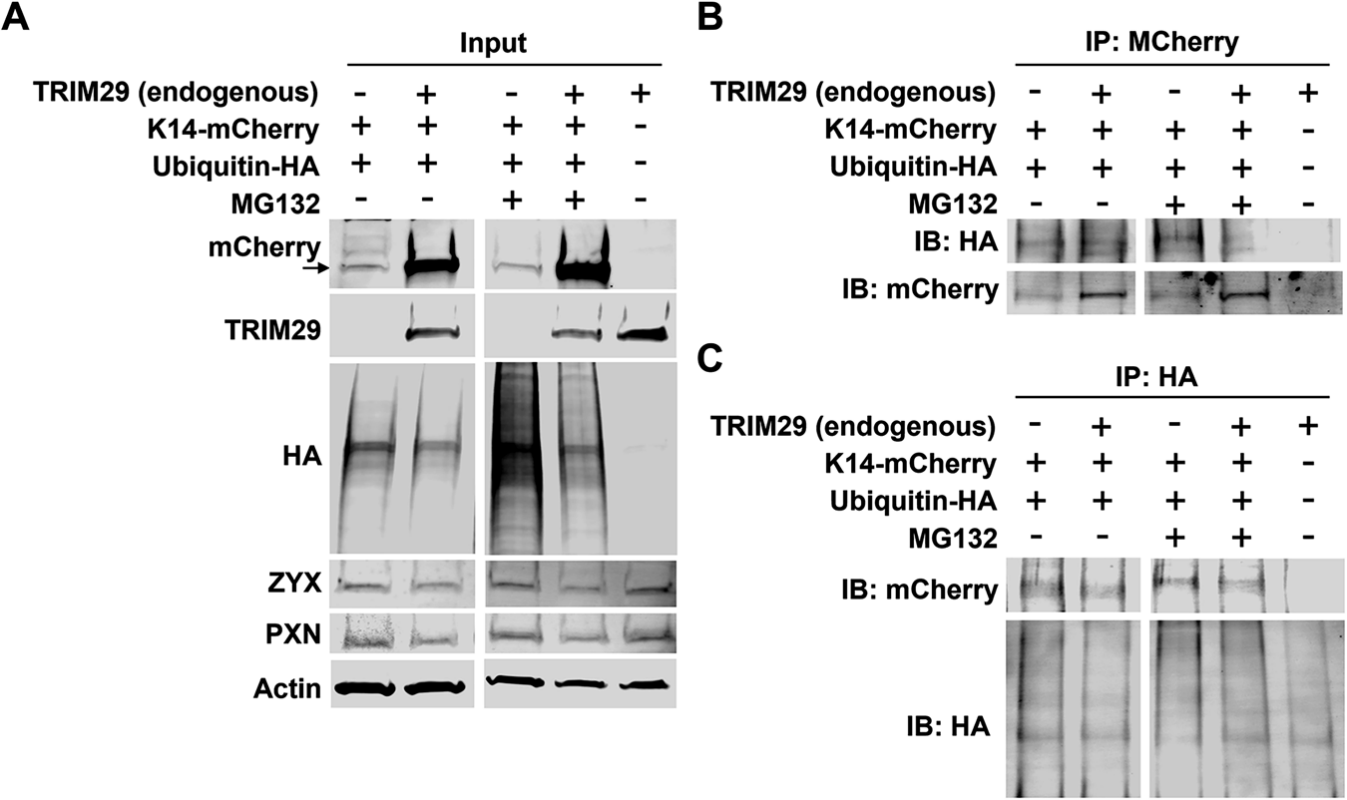
TRIM29 does not modulate K14 proteasomal degradation or ubiquitination. **A** Immunoblots of input for K14-mCherry, TRIM29, ubiquitin-HA, ZYX and PXN in UM-UC14 cells treated with DMSO or MG132 that were used in immunoprecipitations. 30 µg protein/lane. Arrow indicates expected band for K14-mCherry. **B** Immunoprecipitation performed with anti-mCherry antibody shows pull-down of mCherry-K14 and HA-ubiquitin. **C** Immunoprecipitation of HA-ubiquitin resulted in pulldown of K14-mCherry.

TRIM29 has been previously reported to exhibit E3 ubiquitin ligase activity in immune cells and cancer cells, where it plays roles in regulating immune response and cancer progression [20, 21, 26, 27]. TRIM33, a related protein family member, can block ubiquitination and degradation of androgen receptors [28]. To assess whether TRIM29 modulates ubiquitination or proteasomal degradation of K14 in bladder cancer cells, we overexpressed mCherry-K14 and HA-tagged ubiquitin in UM-UC14 cells with and without *TRIM29*-KO. These cells were also treated with the proteasomal inhibitor, MG132. Interestingly, despite its purported role as a ubiquitin ligase, we found that TRIM29 expression actually reduced total ubiquitin levels in UM-UC14 cells (Fig. 3A). Further, while TRIM29 expression did increase protein levels of mCherry-K14, this difference was not significantly impacted by treatment with MG132 (Fig. 3A) suggesting that TRIM29 did not influence K14 proteasomal degradation. To determine if K14 is ubiquitinated and whether TRIM29 influenced this, we next immunoprecipitated using antibodies against mCherry or HA. Immunoblotting for mCherry or HA, respectively, demonstrated that while HA-ubiquitin was detectable in mCherry-K14 IP (Fig. 3B) and mCherry-K14 was detectable in HA IP (Fig. 3C), *TRIM29*-KO did not significantly change ubiquitination levels. These results suggest that while TRIM29 expression does increase K14 protein levels, it does not directly modulate ubiquitination or proteasomal degradation of K14.

### TRIM29 and K14 regulate focal adhesions during invasion

**Fig. 4:**
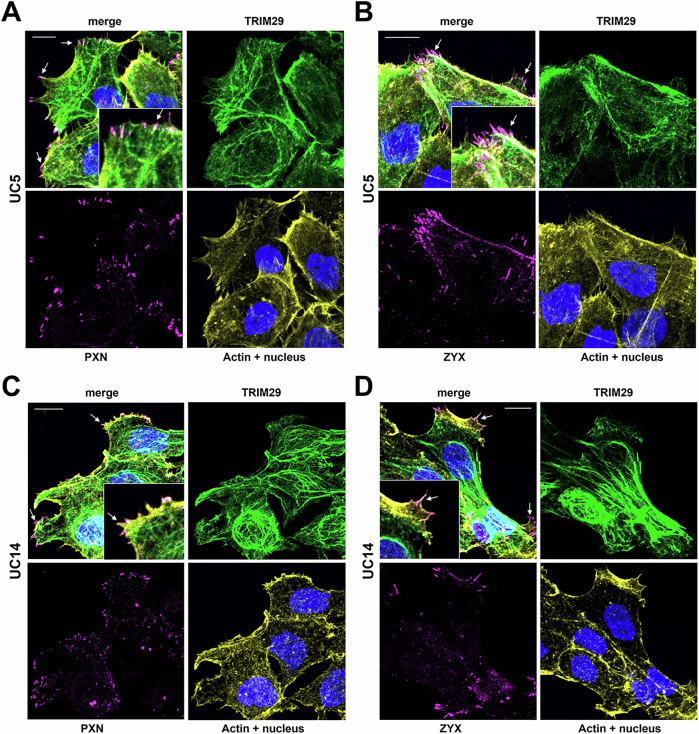
TRIM29+ IF terminate at focal adhesions in migrating cells. TRIM29+ IFs terminate in PXN+ (**A**, **C**) and ZYX+ (**B**, **D**) focal adhesion sites in filipodia and lamellipodia in invasive UM-UC5 (**A**, **B**) and UM-UC14 (**C**, **D**) bladder cancer cells. Inset images show higher magnification view. Scale bar = 10 µm.

**Fig. 5:**
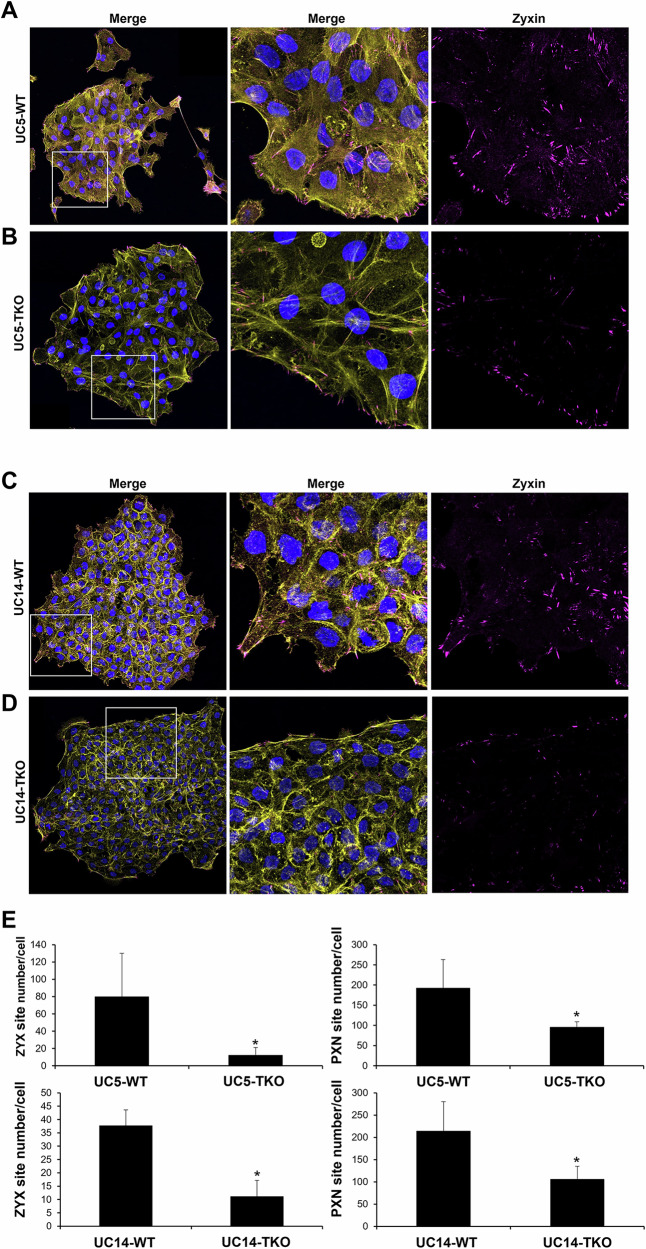
TRIM29 regulates focal adhesion. **A**–**D** *TRIM29*-KO (TKO) reduces ZYX+ focal adhesion sites during UM-UC5 and UM-UC14 bladder cancer spheroid invasion. Magenta: ZYX. Yellow: Actin. Blue: Hoechst 33342+ Nuclei. Scale bar = 10 µm. **E** Quantitative analysis of ZYX+ focal adhesions (UM-UC5-WT, n = 80; UM-UC5-TKO, n = 51; UM-UC14-WT, n = 33; UM-UC14-TKO, n = 18) or PXN+ focal adhesions (UM-UC5-WT, n = 168; UM-UC5-TKO, n = 194; UM-UC14-WT, n = 261; UM-UC14-TKO, n = 340). Data represent the mean ± STD. *p < 0.05 for *t*-test.

Keratin+ IF regulate focal adhesion stability and cellular migration [22], and proteins associated with focal adhesions were pulled down with TRIM29 in UM-UC9 and UM-UC5 cells. Based on these results, we hypothesized that the TRIM29-K14 interaction regulates migration via effects on focal adhesion formation or turnover. To examine this, we performed staining for TRIM29, PXN, zyxin (ZYX, a member of the focal adhesion complex) and actin in our invasive bladder cancer spheroids and found that TRIM29+ IF terminated at PXN+ and ZYX+ FA sites in the migratory bladder cancer cells (Fig. 4A–D). These focal adhesion sites were concentrated in filopodia and lamellipodia (arrows) on the leading edge of migratory cancer cells. To determine if TRIM29 was required for the formation of these FAs during invasion, we examined staining in UM-UC5 and UM-UC14 *TRIM29*-KO cells and found that *TRIM29*-KO significantly reduced ZYX+ and PXN+ focal adhesions (Fig. 5A–E, Supplementary Fig. 5). These results suggest that *TRIM29* is involved in FA regulation during bladder cancer cell migration.

### TRIM29 regulates focal adhesion stability during cancer migration

**Fig. 6:**
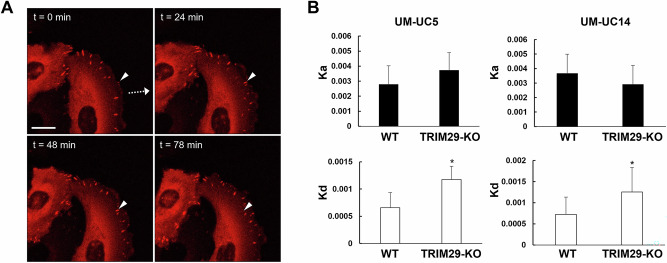
The expression of TRIM29 affects the turnover of focal adhesion sites during cell migration. **A** Timelapse images of UM-UC5 cells expressing mCherry-Paxillin during cell migration. Arrowhead indicates monitored focal adhesion site. Dashed arrow shows the direction of cell movement. Scale bar = 20 µm. **B** *TRIM29* KO had no effect on the rate of assembly (Ka) but increased the rate of disassembly (Kd) of focal adhesion sites in UM-UC5 and UM-UC14 bladder cancer cells (UM-UC5, n = 21; UM-UC14, n = 24). Data represent the mean + STD. *p < 0.05 for *t*-test.

FA presence during cancer cell migration is dependent on the rate of FA formation and dissolution. Since K6+ IF are known to regulate FA formation and resolution [22] and since TRIM29 was found to be part of the focal adhesion protein complex, we hypothesized that TRIM29 might regulate focal adhesion stability during invasion and migration. To examine this, we measured focal adhesion dynamics in WT and *TRIM29*-KO bladder cancer cells expressing a mCherry-tagged PXN construct in a migration assay. As expected, mCherry+ FA were visible at the leading edge of invasive cells during time-lapse microscopy (Fig. 6A). In WT cells, the focal adhesion sites first appeared at the leading edge of cells and became larger as they moved gradually toward the posterior edge of the cells, but the foci in *TRIM29*-KO dispersed more quickly (Supplementary Videos 5-8). To quantify this process, focal adhesion sites from the time-lapse images were randomly selected, mCherry signal was quantified and analysis was performed as previously published [22]. By analyzing the rates of assembly (Ka) and disassembly (Kd) of each focal adhesion site, we found that the rate of disassembly of focal adhesion was higher in *TRIM29*-KO cells, whereas the assembly rate was not different between *TRIM29*-KO and WT (Fig. 6B). These results suggest that TRIM29 regulates FA stability during migration and invasion.

### K14 is required for TRIM29 regulation of FA stability and promotion of bladder cancer migration and invasion

**Fig. 7:**
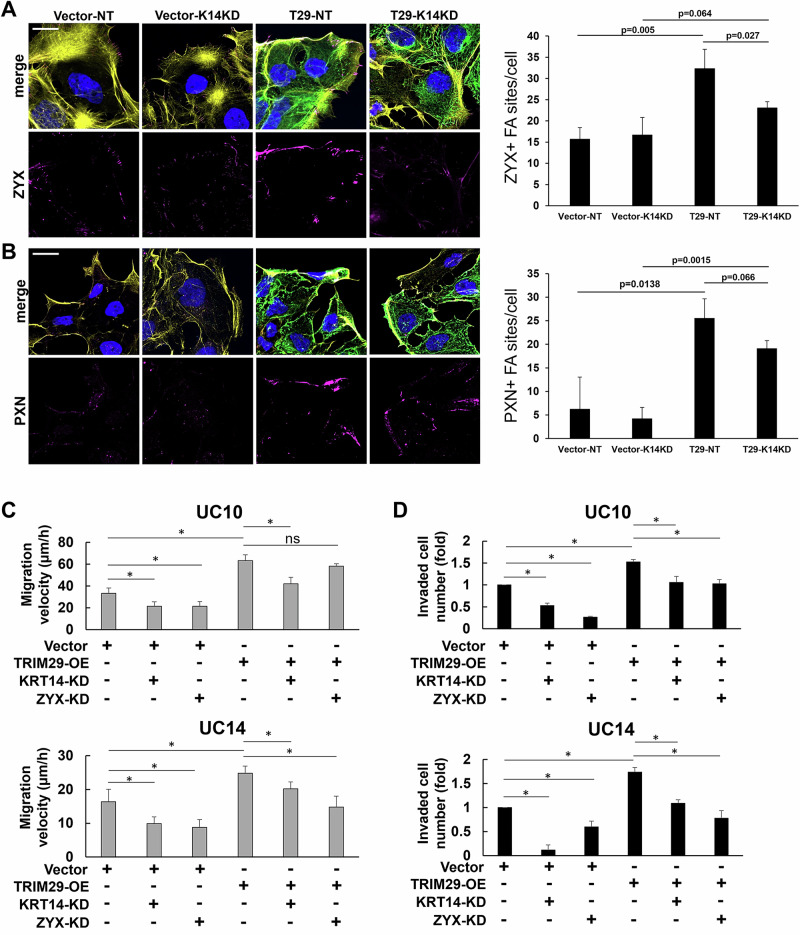
K14 regulates TRIM29-induced focal adhesions and bladder cancer migration. **A**, **B** Re-expression of TRIM29 in the UM-UC14 *TRIM29* KO cell line allows recovery of ZYX+ and PXN+ focal adhesion sites, but this effect is abrogated by K14 KD. Vector: expression vector control. T29: TRIM29 re-expression vector. NT: nontargeting siRNA control. K14KD: siRNA targeting K14. Magenta: ZYX or PXN. Yellow: F-Actin. Green: Reintroduced TRIM29. Blue: Hoechst 33342+ Nuclei. Overexpression of TRIM29 increased migration (**C**) and invasion (**D**) which was abrogated by knockdown of *KRT14* or *ZYX*. Top row: UM-UC10; bottom row: UM-UC14. Vector: empty expression vector control. OE: overexpression. KD: siRNA mediated knockdown. Data represent the mean + STD. *p < 0.01. ns: not significant for one-way ANOVA.

Based on the result that TRIM29 was required to stabilize K14+ IF and focal adhesions during bladder cancer migration and invasion, we hypothesized that TRIM29-mediated stabilization of FA required K14. To test this hypothesis, we knocked down *KRT14* in our *TRIM29*-KO UM-UC14 cells with or without *TRIM29* re-expression (Supplementary Fig. 2) and measured ZYX+ and PXN+ FA sites in migrating cells. *TRIM29* re-expression allowed robust recovery of ZYX+ and PXN+ focal adhesion sites, but this effect on ZYX was abrogated by *KRT14* knockdown (Fig. 7A, B). We also found that the endogenous expression level of ZYX and PXN was not influenced by TRIM29 expression (Fig. 3A). These findings suggest that TRIM29's role in regulating focal adhesions occurs at a post-transcriptional or functional level.

These results suggested that *TRIM29*’s promigratory phenotype would require both *KRT14* and *ZYX*. To test this, we performed RNAi-based gene knock-down of *KRT14* or *ZYX* in UM-UC10 and UM-UC14 bladder cancer cell lines with or without *TRIM29* overexpression (Supplementary Fig. 6). Cell migration was then measured by modified scratch assay, and invasion was measured using a transwell assay. *TRIM29* overexpression promoted increased cancer cell migration and invasion (Fig. 7C, D) [4]. Further, knockdown of *KRT14* or *ZYX* expression blocked *TRIM29*-induced cell migration and transwell invasion (Fig. 7C, D). Taken together, these results demonstrate that K14 is required for TRIM29-mediated stabilization of FA and that both *KRT14* and *ZYX* are required for *TRIM29*-mediated migration and invasion.

## Discussion

**Fig. 8:**
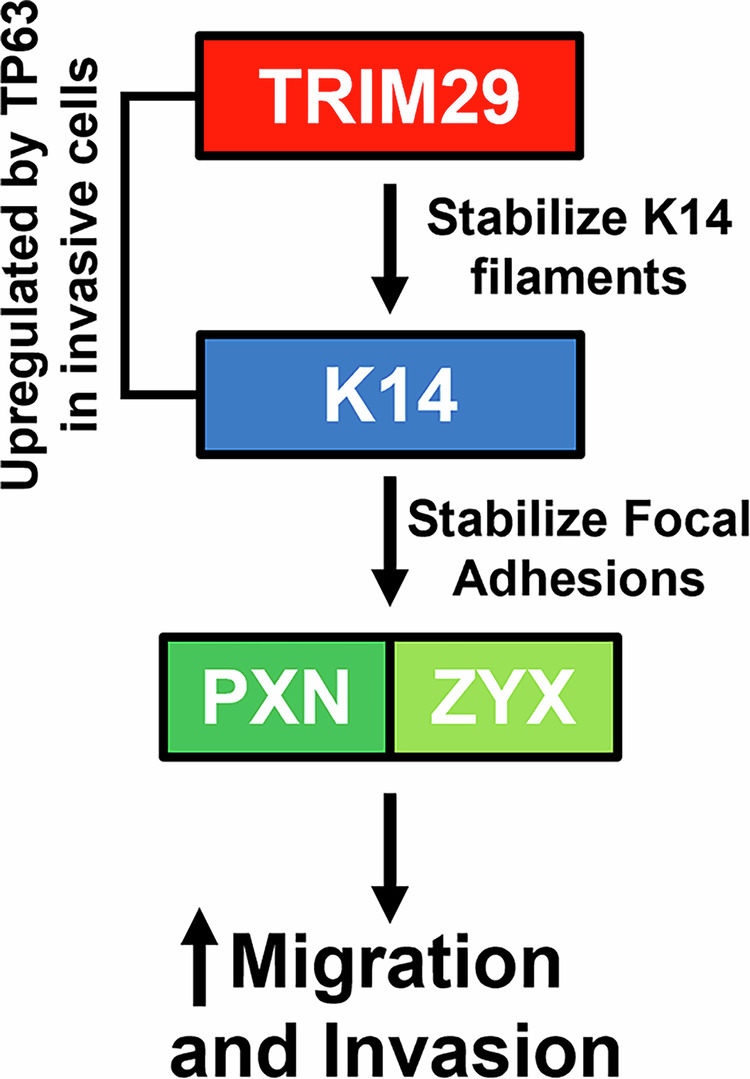
Model of the role of TRIM29, K14 and focal adhesion complexes during bladder cancer migration and invasion.

Invasive progression is the most important biological factor which determines bladder cancer clinical outcomes. We have previously shown that bladder cancer invasion is driven by TP63-mediated upregulation of *TRIM29* and *KRT14* [3]. However, the mechanism whereby TRIM29 and K14 promote invasive progression and metastasis was unclear. In this study, we demonstrate that a primary function of *TRIM29* during invasion is the regulation and promotion of tumor cell migration. Mechanistically, TRIM29 regulates tumor migration by physical association with the K14+ intermediate filaments in invasive bladder cancer cells. Further, the physical association of TRIM29 with K14 and focal adhesion sites appears to stabilize these structures and alter cellular morphology, promoting a pro-invasive, pro-migratory phenotype (Fig. 8). These results establish a new role for TRIM29 in the regulation of cytoskeletal functions and focal adhesion interaction with the extracellular matrix-areas where TRIM29 has not been previously implicated.

The basal molecular subtype of bladder cancer is associated with worse survival and poorer outcomes for patients [29, 30]. Activation of a TP63-mediated basal gene program in tumor cells has been linked to tumor invasion and metastasis in breast and bladder cancers by our group as well as others [3, 4]. Here we demonstrate that this basal gene program which includes upregulation of *TRIM29* and *KRT14*, can occur selectively in invasive bladder cancer cells and specifically promotes cancer cell motility by altering the physical structure of the invasive cell and its attachments to ECM. These results establish novel mechanisms that contribute to the lethal phenotypes seen in basal subtype bladder cancer. They also suggest that treatments which disrupt focal adhesion functions may be an attractive approach for bladder cancer patients with basal subtype tumors that have poor outcomes and for which treatment options are limited.

The role of intermediate filaments and focal adhesion complex proteins in normal cell motility and in cancer cell invasion and metastasis is complex [5, 31–35]. Intermediate filaments are known to interact directly with and regulate the focal adhesion complex [5]. Likewise, the focal adhesion complex, which includes integrins, adaptor proteins such as PXN and ZYX, and kinases like FAK and c-Src, is a known subject to serine and tyrosine phosphorylation which regulates focal adhesion complex formation and dissolution during cell migration [9, 36]. Since we find that TRIM29 modulates focal adhesion dissolution, we hypothesize that it may do so by promoting PXN phosphorylation which has been shown to govern focal adhesion stability [36]. Alternatively, TRIM29 has also been shown to harbor ubiquitin ligase function [20, 21] and it is conceivable that TRIM29 directly ubiquitinates one or multiple components of the focal adhesion complex leading to the phenotypes observed in this study. Despite this notion, we were unable to detect TRIM29-mediated ubiquitination of K14 in multiple experiments.

Effective cell migration requires a tightly regulated cycle of FA assembly and disassembly. Although, in theory, higher rates of FA turnover could promote migration, our focal adhesion dynamics assay revealed that TRIM29 KO cells exhibit faster FA disassembly and reduced migration. While the contribution of FA stability to migration and invasion is complex, we postulate that TRIM29 KO produces destabilized FA which in turn leads to impaired traction force generation, less efficient forward movement and/or reduced c-Src and FAK signaling.

Interestingly, our findings also contrast with a recent study by Tokuchi et al., 2021, which reported that TRIM29 knockdown enhanced migration and invasion in squamous cell carcinoma. This discrepancy may reflect fundamental differences between squamous and urothelial cancers, including cell lineage and regulatory context. Invasive bladder cancer cells often exhibit a basal-like molecular signature, which may uniquely influence TRIM29’s role in cytoskeletal organization and motility. Moreover, Tokuchi et al. identified a TRIM29–FAM83H–keratin 5/14 complex promoting keratin bundling in squamous cells, whereas we did not detect a similar complex in bladder cancer cells. Instead, our results support a model in which TRIM29 influences focal adhesion stability and cytoskeletal integrity, representing a distinct regulatory axis from that observed in squamous epithelium.

High levels of FAK expression often correlate with invasiveness in cancer cells [37], and tumors with high c-Src activity also correlate with malignant potential [38]. In bladder cancer, FAK and c-Src activity have been linked to survival, invasive progression and migration [39–41] and PTEN, a downstream target of TRIM29 [2], also has been shown to impact FAK and AKT phosphorylation [42]. Our results suggest a key function of TRIM29 is to stabilize the focal adhesion complex. It is, therefore, likely this activity also impacts FAK and c-Src signaling, mediating its effect on tumor migration and invasion. If true, this suggests kinase inhibition of FAK or c-Src may be an attractive therapeutic strategy to limit migration/invasion in patients with invasive basal subtype bladder cancers.

## Supplementary information

Supplemental Figure 1

Supplemental Figure 2

Supplemental Figure 3

Supplemental Figure 4

Supplemental Figure 5

Supplemental Figure legends

Video 1

Video 2

Video 3

Video 4

Video 5

Video 6

Video 7

Video 8

Supplmental Table 1

Supplemental Table 2

Supplemental Figure 6

## Data Availability

All data presented in the Results were generated by the authors and are available upon request.
