# Supplementary figures and images for: TRIM29 promotes bladder cancer invasion by regulating the intermediate filament network and focal adhesion
Source: Oncogene. 2025 Sep 4;44(42):4047–57. doi: 10.1038/s41388-025-03557-z (PMC12518127; doi:10.1038/s41388-025-03557-z)

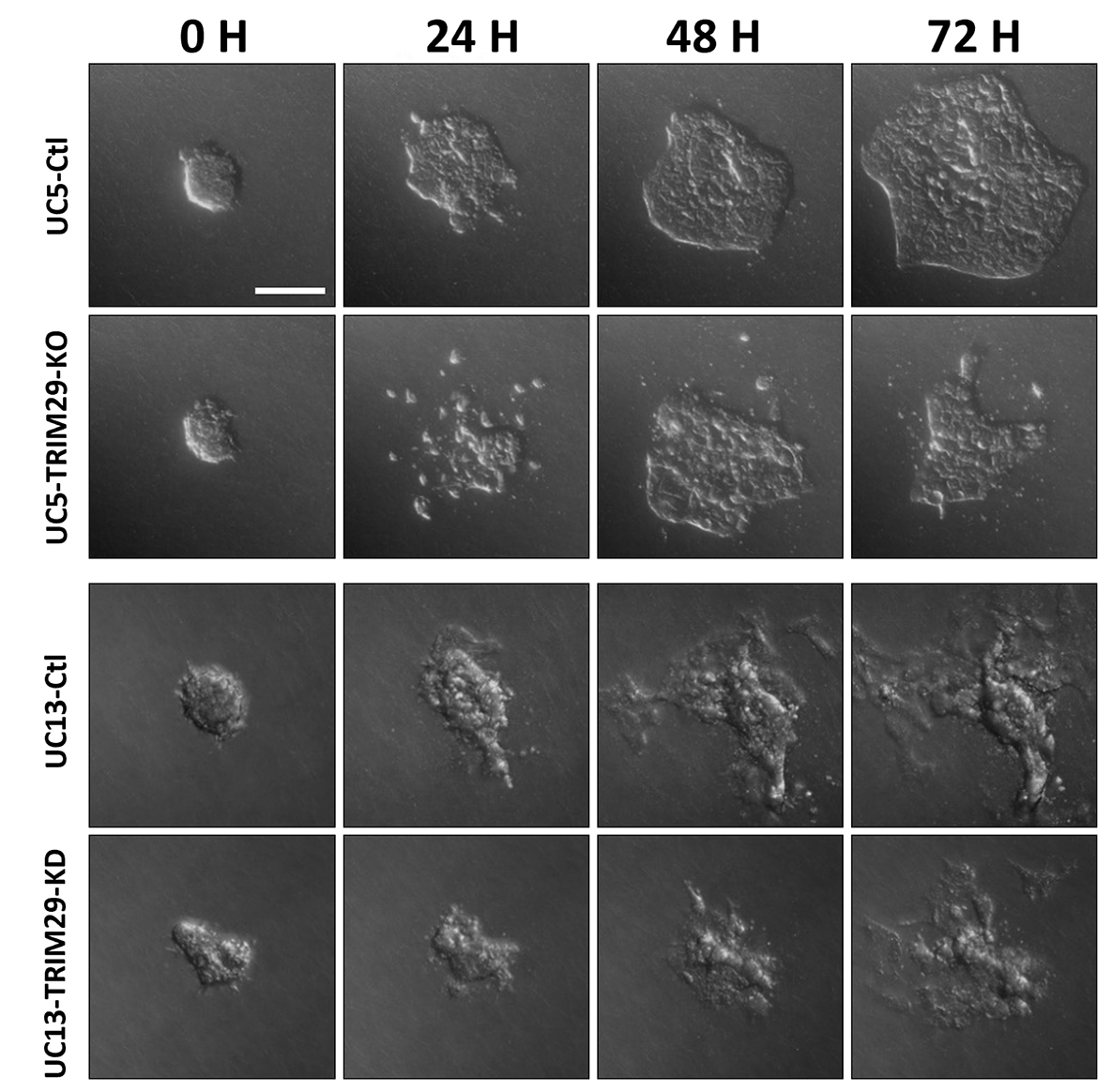

Supplement: Supplementary file 1 — Supplemental Figure 1 [file 41388_2025_3557_MOESM1_ESM.tif]

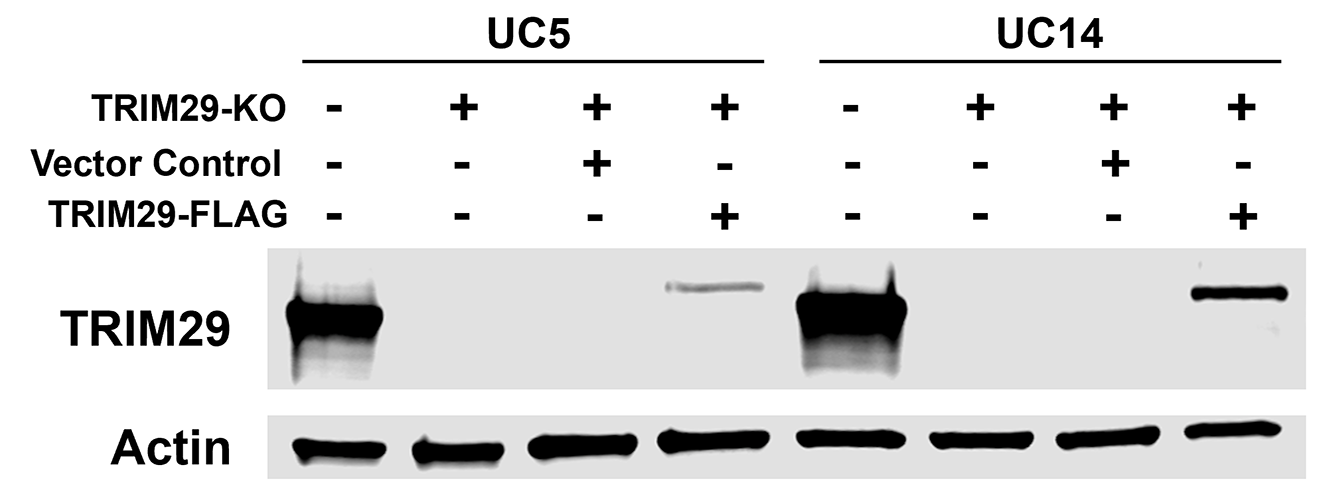

Supplement: Supplementary file 2 — Supplemental Figure 2 [file 41388_2025_3557_MOESM2_ESM.tif]

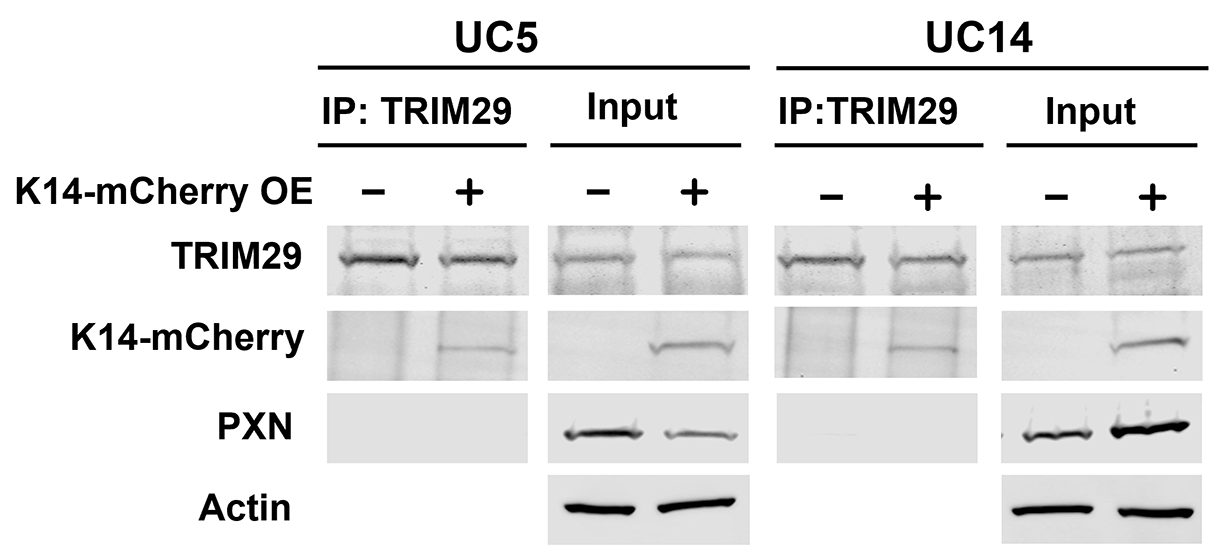

Supplement: Supplementary file 3 — Supplemental Figure 3 [file 41388_2025_3557_MOESM3_ESM.tif]

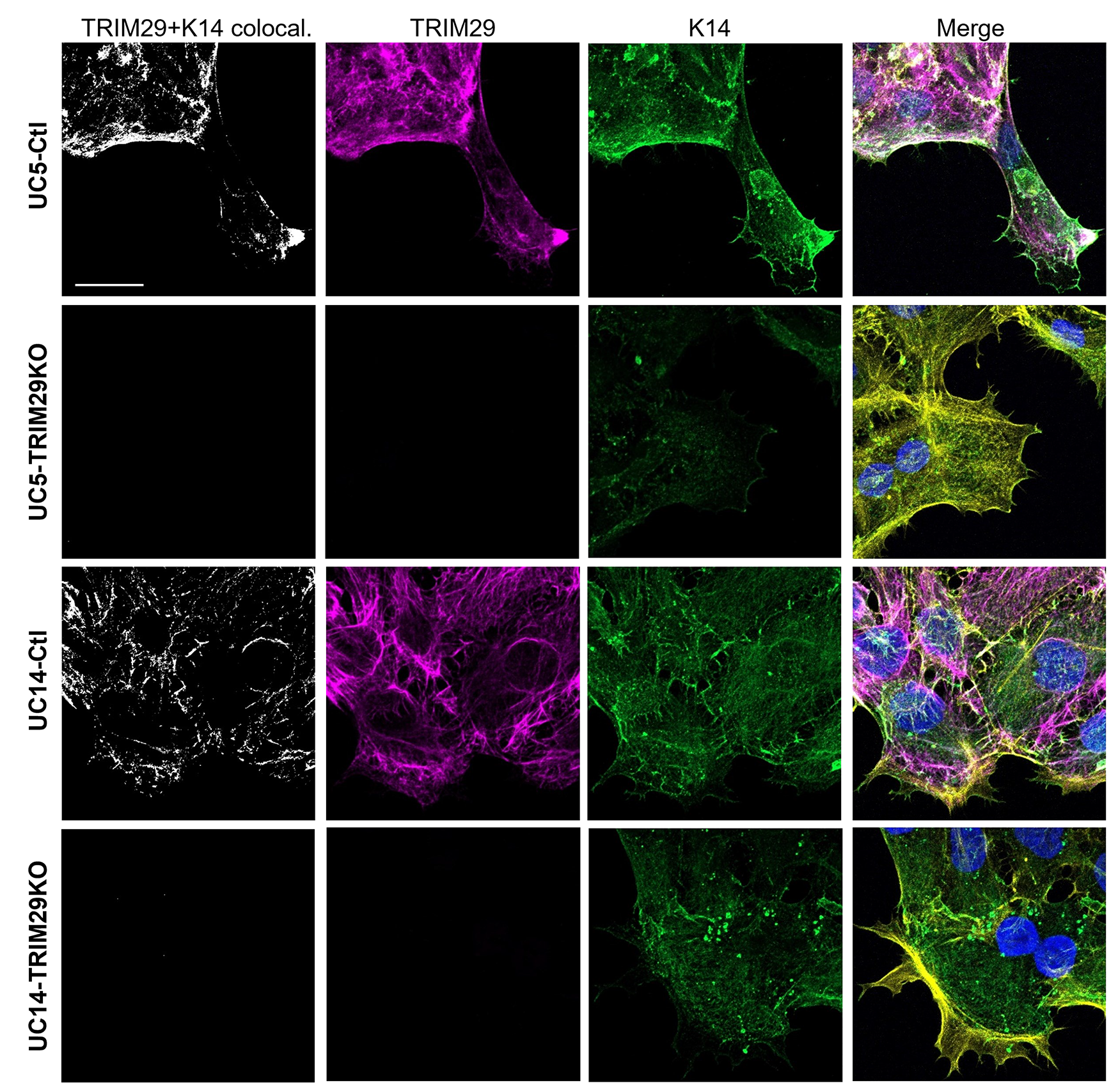

Supplement: Supplementary file 4 — Supplemental Figure 4 [file 41388_2025_3557_MOESM4_ESM.tif]

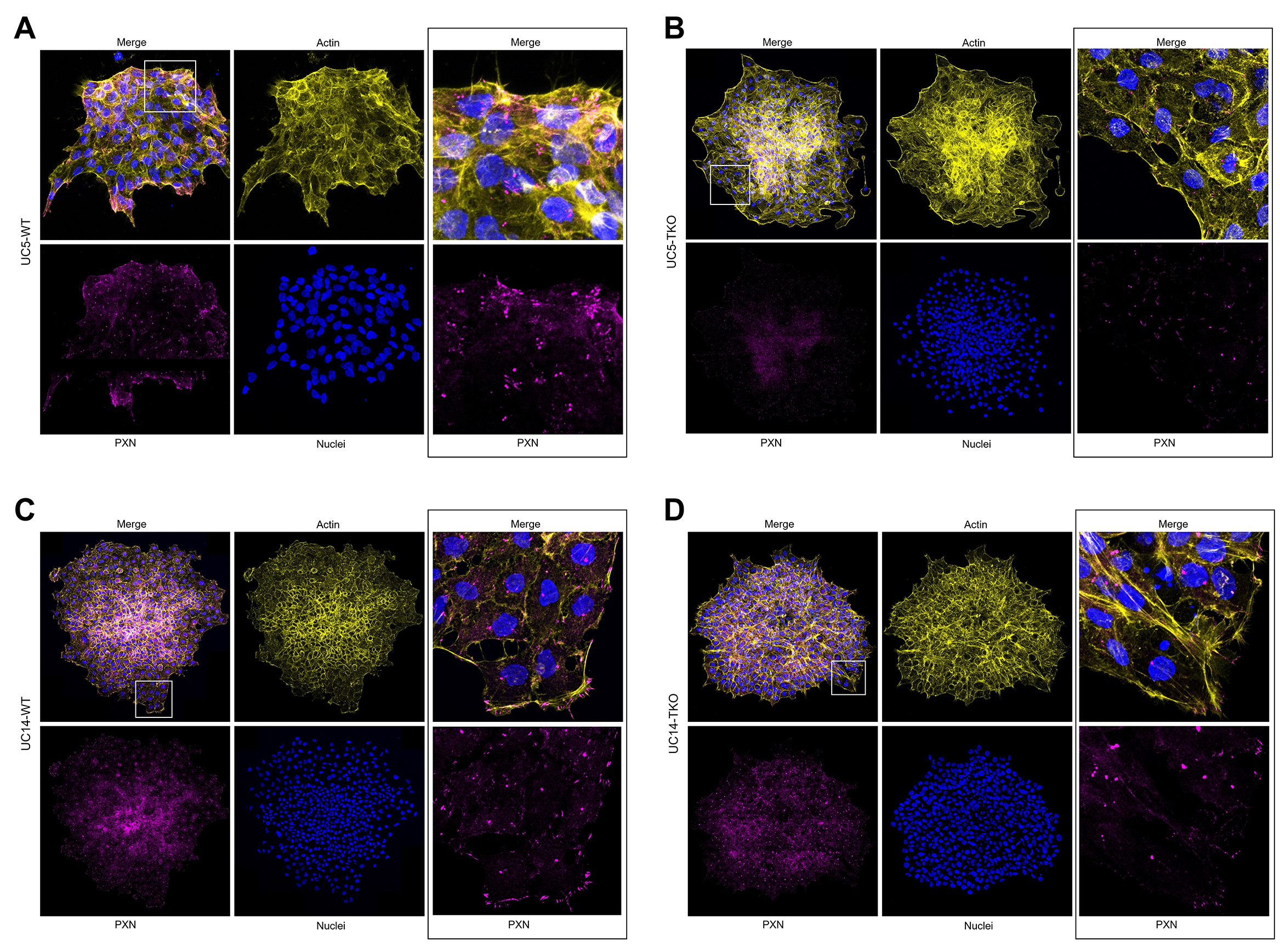

Supplement: Supplementary file 5 — Supplemental Figure 5 [file 41388_2025_3557_MOESM5_ESM.tif]

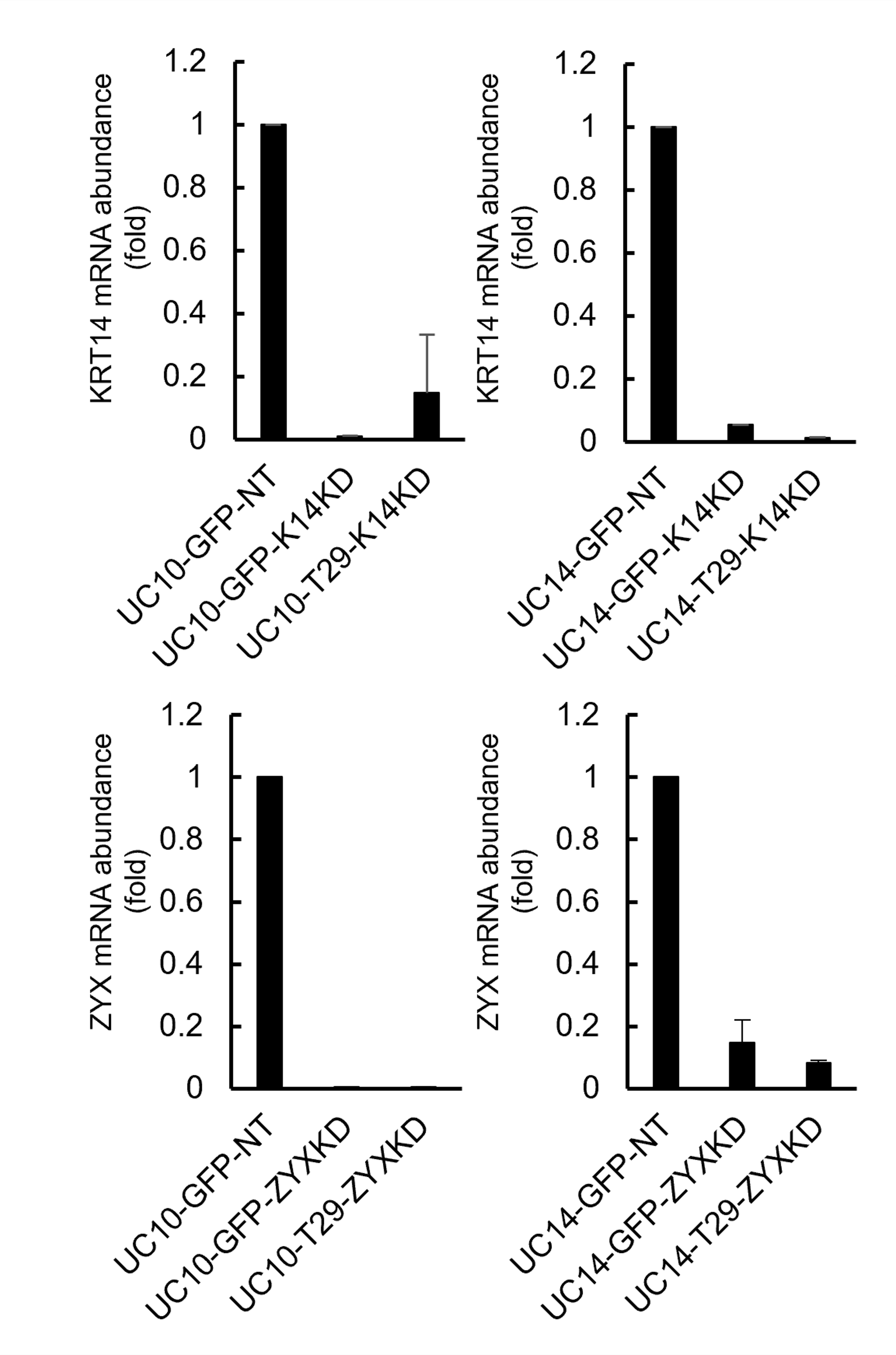

Supplement: Supplementary file 17 — Supplemental Figure 6 [file 41388_2025_3557_MOESM17_ESM.tif]
