# Supplementary material for: TRIM29 promotes bladder cancer invasion by regulating the intermediate filament network and focal adhesion
Source: Oncogene. 2025 Sep 4;44(42):4047–57. doi: 10.1038/s41388-025-03557-z (PMC12518127; doi:10.1038/s41388-025-03557-z)
Supplement: Supplementary file 6 — Supplemental Figure legends [file 41388_2025_3557_MOESM6_ESM.docx]

Supplemental Figure 1. The expression of TRIM29 affects cancer cell invasion. Time-lapse images show the invasion of cancer spheroids in 3D. UC5/13-Ctl = UM-UC5/13 WT, TRIM29-KO = *TRIM29-*knockout, TRIM29-KD = *TRIM29*-knockdown mediated by siRNA. Scale bar = 100 µm.

Supplemental Figure 2. Western blot shows the expression of TRIM29 in UM-UC5 and UM-UC14 cells with or without *TRIM29*-knockout (KO). Re-expression of TRIM29 was performed by expressing TRIM29-FLAG in *TRIM29*-KO cells.

Supplemental Figure 3. Co-immunoprecipitation of TRIM29 with mCherry-tagged K14 confirms a physical association between K14 and TRIM29 in UM-UC5 and UM-UC14.

Supplemental Figure 4. IF staining for TRIM29 and K14 in UM-UC5 and UC14 TRIM29 WT and KO cells. Magenta: TRIM29. Green: K14. Yellow: Actin. Blue: Nuclei. White: colocalization of TRIM29 and K14.

Supplemental Figure 5. TRIM29 regulates focal adhesion. (A-D) Immunofluorescence images demonstrate that KO of *TRIM29* affects the formation of focal adhesion plaques during expansion of bladder cancer spheroids. Focal adhesion sites were represented by immunostaining of Paxillin. WT: wildtype, TKO: *TRIM29*-knockout. Far right panel = higher magnification image of region shown by white box.

Supplemental Figure 6. Gene expression of *KRT14* and *ZYX* in UM-UC10 and UM-UC14 were detected by q-PCR. GFP: overexpression of GFP as control. T29: overexpression of TRIM29, NT: cells transfected with nontargeting control siRNA, K14KD: knockdown of *KRT14* by siRNA transfection, ZYXKD: knockdown of *ZYX* by siRNA transfection. Data represent the mean + STD.

Supplemental Videos

Cells expressing mCherry-labeled K14 were monitored by confocal microscopy. Timelapse images were taken for 24 hours. UM-UC5-Ctl (1), UM-UC5-TKO (2), UM-UC14-Ctl (3), UM-UC14-TKO (4). Scale bar = 50 µm.
