# Supplementary material for: TRIM29 promotes bladder cancer invasion by regulating the intermediate filament network and focal adhesion
Source: Oncogene. 2025 Sep 4;44(42):4047–57. doi: 10.1038/s41388-025-03557-z (PMC12518127; doi:10.1038/s41388-025-03557-z)
Supplement: Supplementary file 16 — Supplemental Table 2 [file 41388_2025_3557_MOESM16_ESM.pptx]

## Slide 1
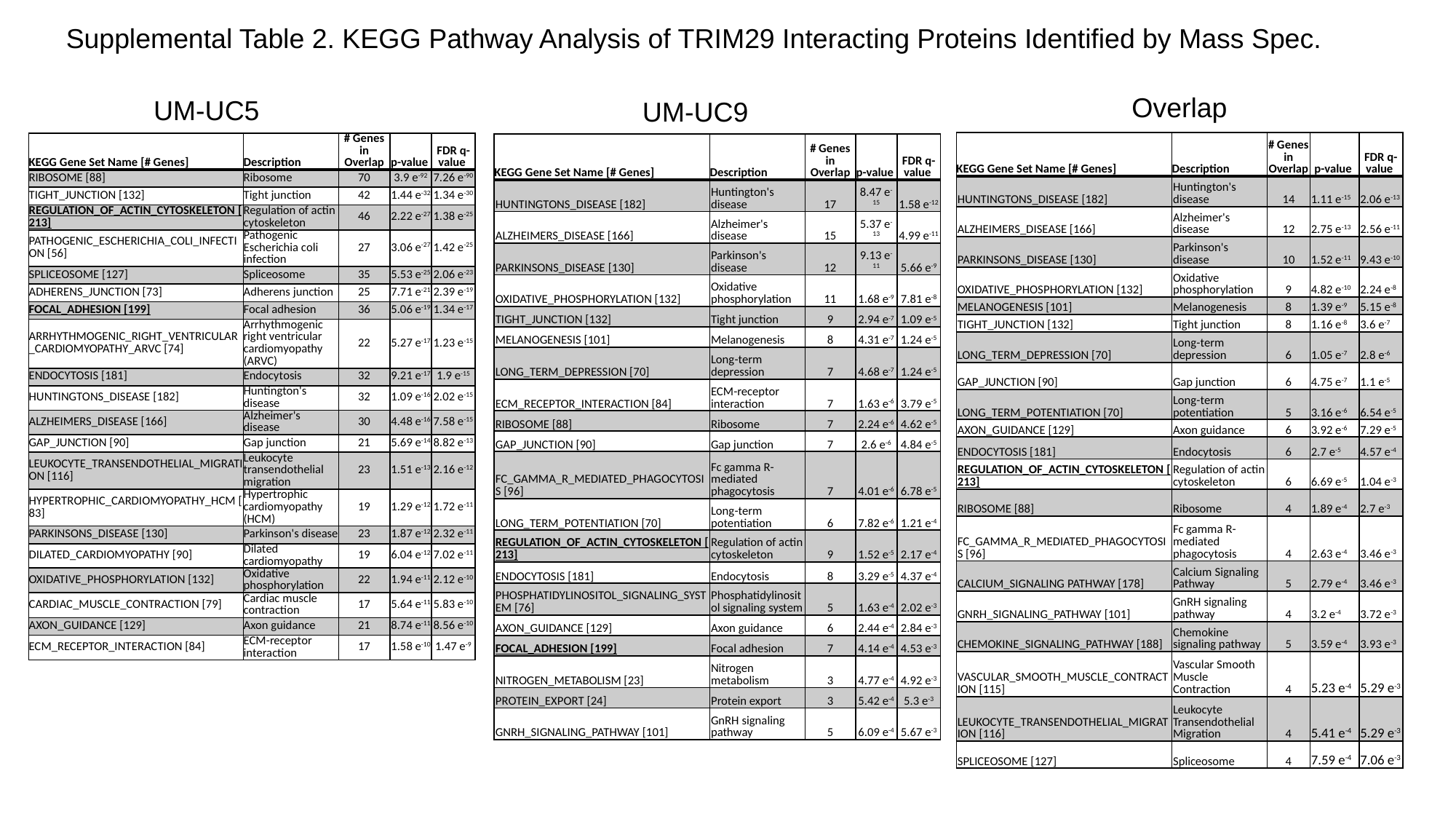

Supplemental Table 2. KEGG Pathway Analysis of TRIM29 Interacting Proteins Identified by Mass Spec.
Overlap
UM-UC5
UM-UC9
| KEGG Gene Set Name [# Genes] | Description | # Genes in Overlap | p-value | FDR q-value |
| --- | --- | --- | --- | --- |
| HUNTINGTONS\_DISEASE [182] | Huntington's disease | 14 | 1.11 e-15 | 2.06 e-13 |
| ALZHEIMERS\_DISEASE [166] | Alzheimer's disease | 12 | 2.75 e-13 | 2.56 e-11 |
| PARKINSONS\_DISEASE [130] | Parkinson's disease | 10 | 1.52 e-11 | 9.43 e-10 |
| OXIDATIVE\_PHOSPHORYLATION [132] | Oxidative phosphorylation | 9 | 4.82 e-10 | 2.24 e-8 |
| MELANOGENESIS [101] | Melanogenesis | 8 | 1.39 e-9 | 5.15 e-8 |
| TIGHT\_JUNCTION [132] | Tight junction | 8 | 1.16 e-8 | 3.6 e-7 |
| LONG\_TERM\_DEPRESSION [70] | Long-term depression | 6 | 1.05 e-7 | 2.8 e-6 |
| GAP\_JUNCTION [90] | Gap junction | 6 | 4.75 e-7 | 1.1 e-5 |
| LONG\_TERM\_POTENTIATION [70] | Long-term potentiation | 5 | 3.16 e-6 | 6.54 e-5 |
| AXON\_GUIDANCE [129] | Axon guidance | 6 | 3.92 e-6 | 7.29 e-5 |
| ENDOCYTOSIS [181] | Endocytosis | 6 | 2.7 e-5 | 4.57 e-4 |
| REGULATION\_OF\_ACTIN\_CYTOSKELETON [213] | Regulation of actin cytoskeleton | 6 | 6.69 e-5 | 1.04 e-3 |
| RIBOSOME [88] | Ribosome | 4 | 1.89 e-4 | 2.7 e-3 |
| FC\_GAMMA\_R\_MEDIATED\_PHAGOCYTOSIS [96] | Fc gamma R-mediated phagocytosis | 4 | 2.63 e-4 | 3.46 e-3 |
| CALCIUM\_SIGNALING PATHWAY [178] | Calcium Signaling Pathway | 5 | 2.79 e-4 | 3.46 e-3 |
| GNRH\_SIGNALING\_PATHWAY [101] | GnRH signaling pathway | 4 | 3.2 e-4 | 3.72 e-3 |
| CHEMOKINE\_SIGNALING\_PATHWAY [188] | Chemokine signaling pathway | 5 | 3.59 e-4 | 3.93 e-3 |
| VASCULAR\_SMOOTH\_MUSCLE\_CONTRACTION [115] | Vascular Smooth Muscle Contraction | 4 | 5.23 e-4 | 5.29 e-3 |
| LEUKOCYTE\_TRANSENDOTHELIAL\_MIGRATION [116] | Leukocyte Transendothelial Migration | 4 | 5.41 e-4 | 5.29 e-3 |
| SPLICEOSOME [127] | Spliceosome | 4 | 7.59 e-4 | 7.06 e-3 |
| KEGG Gene Set Name [# Genes] | Description | # Genes in Overlap | p-value | FDR q-value |
| --- | --- | --- | --- | --- |
| RIBOSOME [88] | Ribosome | 70 | 3.9 e-92 | 7.26 e-90 |
| TIGHT\_JUNCTION [132] | Tight junction | 42 | 1.44 e-32 | 1.34 e-30 |
| REGULATION\_OF\_ACTIN\_CYTOSKELETON [213] | Regulation of actin cytoskeleton | 46 | 2.22 e-27 | 1.38 e-25 |
| PATHOGENIC\_ESCHERICHIA\_COLI\_INFECTION [56] | Pathogenic Escherichia coli infection | 27 | 3.06 e-27 | 1.42 e-25 |
| SPLICEOSOME [127] | Spliceosome | 35 | 5.53 e-25 | 2.06 e-23 |
| ADHERENS\_JUNCTION [73] | Adherens junction | 25 | 7.71 e-21 | 2.39 e-19 |
| FOCAL\_ADHESION [199] | Focal adhesion | 36 | 5.06 e-19 | 1.34 e-17 |
| ARRHYTHMOGENIC\_RIGHT\_VENTRICULAR\_CARDIOMYOPATHY\_ARVC [74] | Arrhythmogenic right ventricular cardiomyopathy (ARVC) | 22 | 5.27 e-17 | 1.23 e-15 |
| ENDOCYTOSIS [181] | Endocytosis | 32 | 9.21 e-17 | 1.9 e-15 |
| HUNTINGTONS\_DISEASE [182] | Huntington's disease | 32 | 1.09 e-16 | 2.02 e-15 |
| ALZHEIMERS\_DISEASE [166] | Alzheimer's disease | 30 | 4.48 e-16 | 7.58 e-15 |
| GAP\_JUNCTION [90] | Gap junction | 21 | 5.69 e-14 | 8.82 e-13 |
| LEUKOCYTE\_TRANSENDOTHELIAL\_MIGRATION [116] | Leukocyte transendothelial migration | 23 | 1.51 e-13 | 2.16 e-12 |
| HYPERTROPHIC\_CARDIOMYOPATHY\_HCM [83] | Hypertrophic cardiomyopathy (HCM) | 19 | 1.29 e-12 | 1.72 e-11 |
| PARKINSONS\_DISEASE [130] | Parkinson's disease | 23 | 1.87 e-12 | 2.32 e-11 |
| DILATED\_CARDIOMYOPATHY [90] | Dilated cardiomyopathy | 19 | 6.04 e-12 | 7.02 e-11 |
| OXIDATIVE\_PHOSPHORYLATION [132] | Oxidative phosphorylation | 22 | 1.94 e-11 | 2.12 e-10 |
| CARDIAC\_MUSCLE\_CONTRACTION [79] | Cardiac muscle contraction | 17 | 5.64 e-11 | 5.83 e-10 |
| AXON\_GUIDANCE [129] | Axon guidance | 21 | 8.74 e-11 | 8.56 e-10 |
| ECM\_RECEPTOR\_INTERACTION [84] | ECM-receptor interaction | 17 | 1.58 e-10 | 1.47 e-9 |
| KEGG Gene Set Name [# Genes] | Description | # Genes in Overlap | p-value | FDR q-value |
| --- | --- | --- | --- | --- |
| HUNTINGTONS\_DISEASE [182] | Huntington's disease | 17 | 8.47 e-15 | 1.58 e-12 |
| ALZHEIMERS\_DISEASE [166] | Alzheimer's disease | 15 | 5.37 e-13 | 4.99 e-11 |
| PARKINSONS\_DISEASE [130] | Parkinson's disease | 12 | 9.13 e-11 | 5.66 e-9 |
| OXIDATIVE\_PHOSPHORYLATION [132] | Oxidative phosphorylation | 11 | 1.68 e-9 | 7.81 e-8 |
| TIGHT\_JUNCTION [132] | Tight junction | 9 | 2.94 e-7 | 1.09 e-5 |
| MELANOGENESIS [101] | Melanogenesis | 8 | 4.31 e-7 | 1.24 e-5 |
| LONG\_TERM\_DEPRESSION [70] | Long-term depression | 7 | 4.68 e-7 | 1.24 e-5 |
| ECM\_RECEPTOR\_INTERACTION [84] | ECM-receptor interaction | 7 | 1.63 e-6 | 3.79 e-5 |
| RIBOSOME [88] | Ribosome | 7 | 2.24 e-6 | 4.62 e-5 |
| GAP\_JUNCTION [90] | Gap junction | 7 | 2.6 e-6 | 4.84 e-5 |
| FC\_GAMMA\_R\_MEDIATED\_PHAGOCYTOSIS [96] | Fc gamma R-mediated phagocytosis | 7 | 4.01 e-6 | 6.78 e-5 |
| LONG\_TERM\_POTENTIATION [70] | Long-term potentiation | 6 | 7.82 e-6 | 1.21 e-4 |
| REGULATION\_OF\_ACTIN\_CYTOSKELETON [213] | Regulation of actin cytoskeleton | 9 | 1.52 e-5 | 2.17 e-4 |
| ENDOCYTOSIS [181] | Endocytosis | 8 | 3.29 e-5 | 4.37 e-4 |
| PHOSPHATIDYLINOSITOL\_SIGNALING\_SYSTEM [76] | Phosphatidylinositol signaling system | 5 | 1.63 e-4 | 2.02 e-3 |
| AXON\_GUIDANCE [129] | Axon guidance | 6 | 2.44 e-4 | 2.84 e-3 |
| FOCAL\_ADHESION [199] | Focal adhesion | 7 | 4.14 e-4 | 4.53 e-3 |
| NITROGEN\_METABOLISM [23] | Nitrogen metabolism | 3 | 4.77 e-4 | 4.92 e-3 |
| PROTEIN\_EXPORT [24] | Protein export | 3 | 5.42 e-4 | 5.3 e-3 |
| GNRH\_SIGNALING\_PATHWAY [101] | GnRH signaling pathway | 5 | 6.09 e-4 | 5.67 e-3 |
